# Atypical Enostoses—Series of Ten Cases and Literature Review

**DOI:** 10.3390/medicina56100534

**Published:** 2020-10-13

**Authors:** Thomas Bedard, Mujtaba Mohammed, Serenella Serinelli, Timothy A. Damron

**Affiliations:** 1 Department of Orthopedic Surgery, SUNY Upstate Medical University, Syracuse, New York, NY 13210, USA; bedardt@upstate.edu; 2 Department of Radiology, SUNY Upstate Medical University, Syracuse, New York, NY 13210, USA; mohammem@upstate.edu; 3 Department of Pathology, SUNY Upstate Medical University, Syracuse, New York, NY 13210, USA; serinels@upstate.edu

**Keywords:** bone island, enostosis, osteopoikilosis, osteoid osteoma, CT attenuation

## Abstract

Bone islands (BI; enostoses) may be solitary or occur in the setting of osteopoikilosis (multiple bone islands) and are sometimes associated with Gardner’s Syndrome (osteopoikilosis and colonic polyposis). Characteristic features of bone islands are (1) absence of pain or local tenderness, (2) typical radio dense central appearance with peripheral radiating spicules (rose thorn), (3) Mean CT (computerized tomography) attenuation values above 885 Hounsfield units (HU) (4) absence of uptake on bone scan and (5) radiographic stability over time. However, when enostoses display atypical features of pain, unusual radiographic appearance, aberrant HU, increased radiotracer uptake, and/or enlargement, they can be difficult to differentiate from more sinister bony lesions such as osteoblastic metastasis, low grade central osteosarcoma, osteoid osteoma and osteoblastoma. In this retrospective case series, the demographic, clinical, radiographic, treatment and outcome for ten patients with eleven atypical bone islands (ABI) are presented, some showing associated pain (5), some with atypical radiographic appearance (3), some with increased activity on BS (4), some with documented enlargement over time (7), one with abnormal CT attenuation value, some in the setting of osteopoikilosis (2), one in the setting of Gardner’s Syndrome and one osteoid osteoma simulating a bone island. This series represents the spectrum of presentations of ABI. Comprehensive review of the literature reveals that the previous largest series of ABI showing enlargement as the atypical feature was in younger patients with jaw BI. Hence, this represents one of the largest series reported of ABI of all types in adults.

## 1. Introduction

Enostosis, also known as bone island (BI), is a common benign osseous lesion that consists of a focus of compact (cortical) bone within cancellous (spongy) bone [1,2]. Characteristically, enostoses are asymptomatic, “cold” on bone scintigraphy and stable metabolically with scarce evidence of documented growth [2,3,4,5,6]. However, when enostoses display atypical features of pain, increased radiotracer uptake, and/or enlargement, they can be difficult to differentiate from more sinister bony lesions such as osteoblastic metastasis, low-grade central osteosarcoma, osteoid osteoma and osteoblastoma.

CT attenuation is defined as the radiodensity of each material and is expressed in HU, in which the radiodensity of distilled water at the standard pressure and temperature is defined as 0 Hounsfield units (HU) [7]. Each tissue has unique CT attenuation values, although the values vary by tissue components [7]. Recently, mean CT attenuation thresholds have been proposed to differentiate BI from untreated osteoblastic metastasis with a mean of 885 HU serving as the cutoff above which BI is favored and below which a metastatic lesion is the favored diagnosis [2,3].

Here, we report ten cases of atypical enostoses involving the pelvis, acetabulum, femur and spine to increase awareness of diverse presentations and illuminate the diagnostic challenge they can pose.

## 2. Materials and Methods

A retrospective chart review case series was performed including patients from the senior author’s orthopedic oncology database, according to the diagnosis of bone island. This study was deemed Institutional Review Board exempt by SUNY (State University of New York) Upstate Medical University.

The inclusion criteria for this study included patients with a bone island or multiple bone islands that exhibited atypical features on history, physical examination or on imaging. These features included pain, tenderness to palpation, enlargement over time and atypical radiologic appearance. The exclusion criteria included typical bone islands not associated with the aforementioned criteria and those that were later found to represent a malignant diagnosis such as metastatic carcinoma or low-grade central osteosarcoma. This query generated cases with both clinically and histologically confirmed bone islands.

The records of 10 patients with atypical bone island(s) (ABI) were examined through Electronic Medical Records (EMR) chart review, including office notes, operative reports, radiologic interpretations and histologic results. For each ABI, location, tenderness to palpation (TTP), pain corresponding to the site of the lesion, growth, imaging findings, mean CT attenuation (measured in HU), uptake on bone scintigraphy, number of bone lesions, management and diagnosis were obtained. Furthermore, the age and gender of each patient was also recorded.

The imaging for each patient was obtained directly through the EMR or through correspondence with other institutions where the imaging was stored. CT scans were analyzed for HU by a board certified radiologist. Postoperative CT scans were excluded from analysis where applicable.

Two of the most unusual cases were discussed in detail. Our first unusual case presented was selected because an ABI (enlargement) arose in the setting of osteopoikilosis. We juxtaposed this with our second unusual case in which an ABI (enlargement) and osteoid osteoma mimicking ABI (TTP, pain) were diagnosed via biopsy in a patient with osteopoikilosis. This demonstrates that other bone lesions may occur in the setting of osteopoikilosis, so atypical features should cause justifiable concern and appropriate action.

Literature review was conducted through PubMed and Google Scholar databases. Our search terms included “bone island”, “enostosis”, “enlarging enostosis” and “CT attenuation”. All resulting articles, including retrospective case series, case reports and review articles were included in the literature review.

## 3. Results

**Figure 1:**
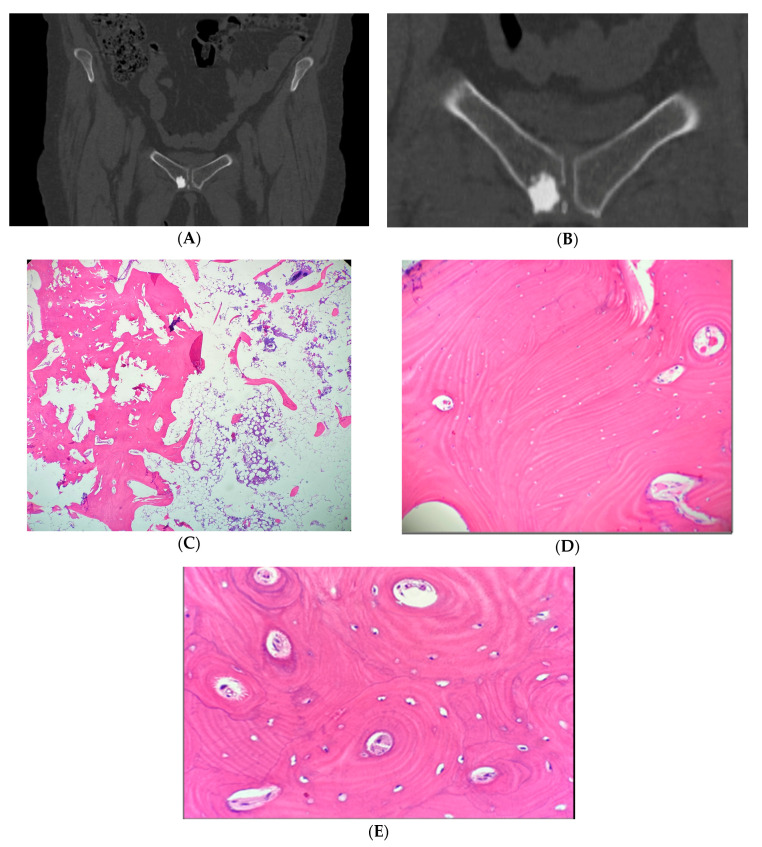
(**A**,**B**). Case 4. Pelvic CT shows sclerotic right pubic bone lesion with cortical erosion. (**A**) Pelvic coronal CT shows a 16 mm sclerotic bone lesion in the right pubic bone near the pubic symphysis. (**B**) Magnification of the sclerotic focus near the pubic symphysis demonstrates cortical erosion, which was felt to be atypical for BI. As such, biopsy was performed, confirming the diagnosis. (**C**–**E**) Case 4. Hematoxylin and Eosin (H&E) stain of right pubic bone biopsy, confirming a diagnosis of atypical bone island. (**C**) Magnification 4× (low power view) of the right pubic bone, showing transition between a bone island composed of cortical type bone (on the left) and the adjacent trabecular bone (on the right). The bone marrow is predominantly fatty with scattered islands of hematopoietic cells. (**D**) Magnification 20× (high power view) of the bone island displaying dense lamellar bone with small osteocytes. (**E**) Magnification 40× (high power view) of the bone island showing numerous haversian-like systems.

**Figure 2:**
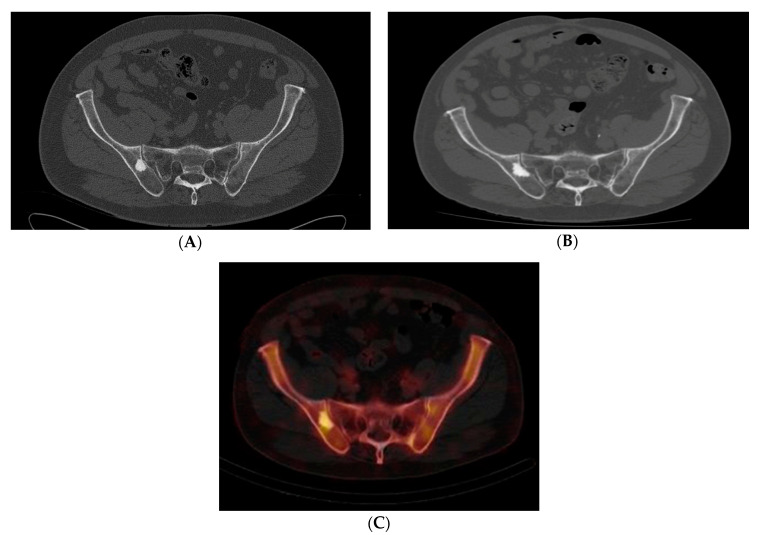
(**A**–**C**) Case 3. Pelvic axial CT shows an enlarged lesion 10 years after original discovery, which also demonstrated mild radiotracer uptake. (**A**) Initial pelvic axial CT shows an 18 mm sclerotic lesion in the right ilium near the SI joint. (**B**) Repeat pelvic CT 10 years later shows enlargement of the lesion to 27 mm. (**C**) Axial SPECT-CT Tc-99m bone scan demonstrated mildly increased radiotracer in the right iliac bone correlating with the region of the patient’s enlarging bone lesion. Although low-grade central osteosarcoma or osteoblastoma could not be ruled out, we believed atypical BI was the most likely diagnosis. The patient opted for observation rather than bone biopsy. There has been no routine follow up visits over the last 10 months.

Ten patients with eleven atypical bone islands (ABI) are presented. Among the ten patients, five showed associated pain, seven demonstrated interval enlargement, two had an atypical imaging appearance including one with cortical erosion (Figure 1A,B) and five showed increased activity on BS including one with concurrent enlargement (Figure 2A–C). Mean CT attenuation values ranged from 528 to 1375 Hounsfield Units (HU) with only one BI falling below the proposed 885 HU cutoff [2,3]. Most bone islands were found in the pelvis (7) followed by the femur (3) and vertebrae (1). Most patients were female (eight of the ten cases). Average age was 58.9 years (range—43–87 years). Diagnosis was established clinically in four lesions and by biopsy in seven others. Two atypical BI occurred in the setting of osteopoikilosis. In one of those cases, an atypical area was discovered to be an osteoid osteoma rather than BI. Two of the more unusual cases are presented in detail.

### 3.1. Case 1

**Figure 3:**
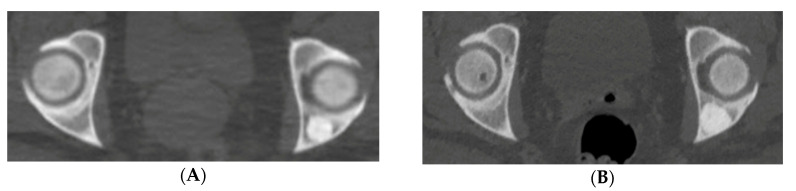

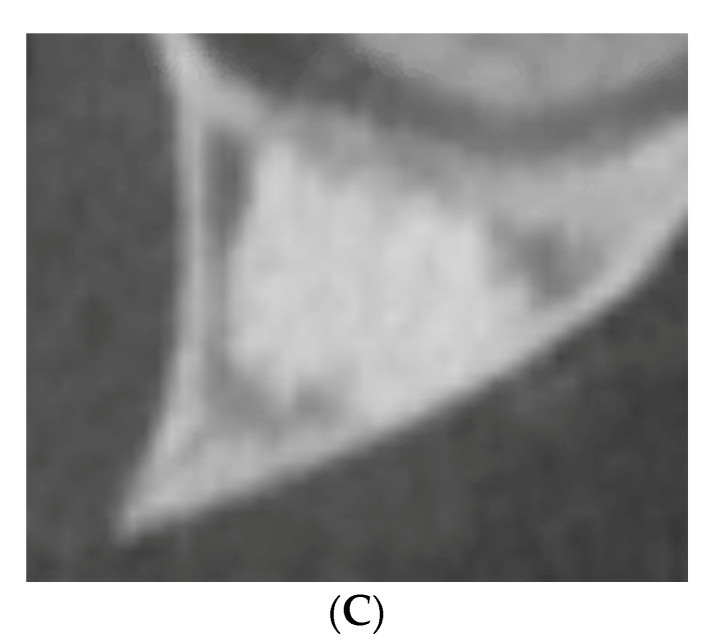
(**A**–**C**) Case 1. Pelvic axial CT shows an enlarged lesion 5 years after original discovery, (**A**) Initial CT displays a 16 mm sclerotic focus in the left posterior acetabulum. (**B**) Repeat CT demonstrates growth of the bone lesion to 21 mm, central radiodensity and peripheral “rose thorn” appearance. (**C**) Higher magnification emphasizes characteristic features. Despite its enlargement, it was felt to be otherwise typical for BI, so observation was recommended.

A 46-year-old Caucasian female with a past medical history of multiple incidentally noted bone lesions of the pelvis and acetabulum presented after an abdominal/pelvis computed tomography (CT) scan revealed enlargement of one lesion. The posterior iliac lesion had increased from 16 to 21 mm diameter over the previous four years (Figure 3A,B). It had the appearance of a sclerotic, rose thorn appearing (Figure 3C) lesion otherwise typical of BI. Routine mammograms had been negative and there was no personal history of any cancer.

Physical examination revealed a healthy appearing woman with full painless passive hip range of motion (ROM). There was no appreciable mass or tenderness to palpation over the groin, greater trochanter (GT) bursa, ischial tuberosity or ilium. Stinchfield, impingement and flexion/abduction/external rotation (FABER) testing were all negative.

A baseline bone scintigraphy (BS) was ordered, which did not exhibit increased uptake in any of the sclerotic bony lesions. We diagnosed the patient clinically with osteopoikilosis with one atypical enlarging giant bone island based on the incidental discovery, asymptomatic clinical course, presence of multiple bilateral radiodense areas in the pelvis consistent with other bone islands, classic radiographic appearance of rose thorn radiating spicules around the largest of the lesions, absence of radiotracer uptake on bone scan and mean CT attenuation value of 998 HU (with the typical attenuation threshold value for BI being >885). Given that these lesions are benign and very slow growth had been documented, she was advised to follow up if symptoms developed. There has been no follow up from the patient over the last 18 months, which further supports, our presumed diagnosis.

### 3.2. Case 2

Five years after initial presentation, a 43-year-old female with a past medical history of colonic polyposis, remote history of unknown benign tumor of the right femur and osteopoikilosis returned with left sided pelvic pain. Prior BS (bone scan) showed no uptake in any of her lesions. There was a 50% pain reduction after taking NSAIDs. Physical exam revealed mild tenderness to palpation (TTP) over the left posterior superior iliac spine (PSIS). Provocative testing did not produce groin pain on either side.

**Figure 4:**
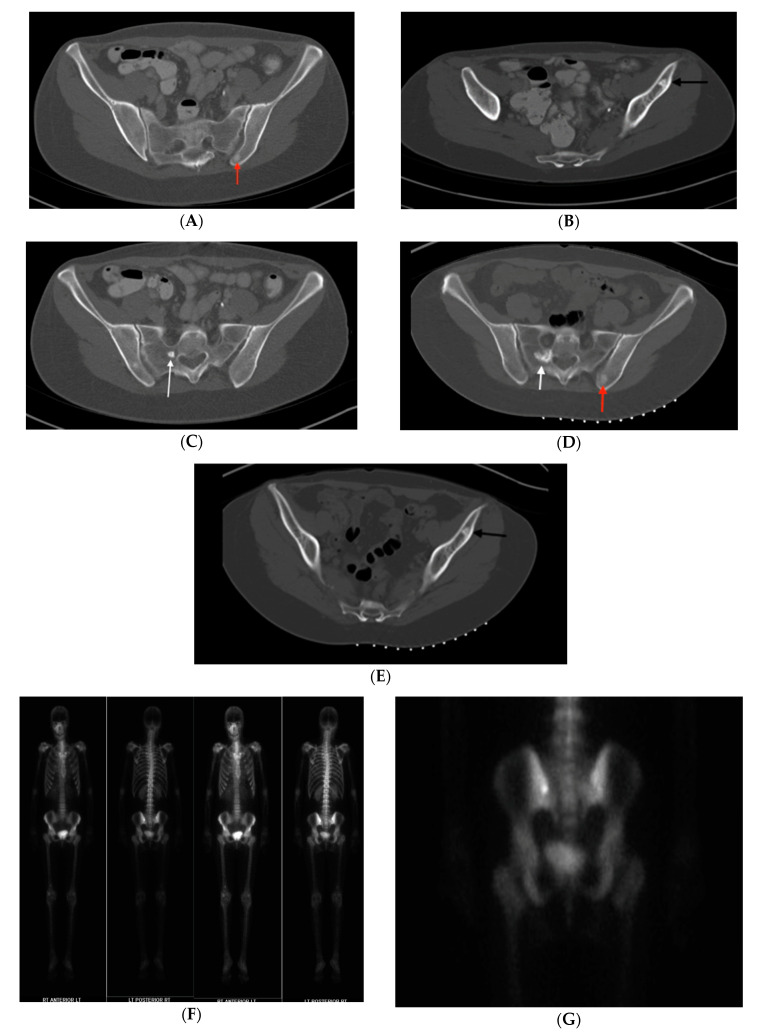

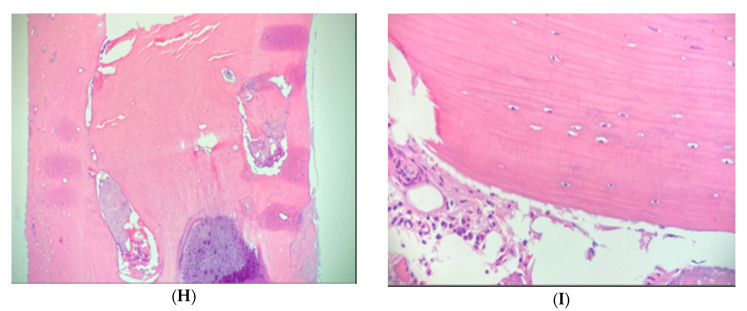
(**A**–**I**) Case 2. Pelvic axial CT shows enlargement, atypical central radiolucency and osteoid osteoma mimicking BI in a patient with osteopoikilosis. (**A**–**C**) Initial CT shows a right sacral lesion (white arrow) measuring 8 mm, left anterior iliac centrally radiolucent less sclerotic lesion (black arrow) and sclerotic bone lesion in the left posterior ilium (red arrow) near the SI joint. (**D**,**E**) Repeat CT 5-years later shows enlargement to 18 mm in right sacrum (white arrow), stable centrally radiolucent left anterior iliac lesion (black arrow) and left posterior iliac lesion (red arrow) correlating with the patient’s pain, TTP (tenderness to palpation) and increased BS (bone scan) uptake. (**F**) NM whole body bone scintigraphy demonstrating increased uptake in the left posterior ilium adjacent to the SI joint. (**G**) Higher magnification emphasizes increased uptake. Given the atypical features, biopsies of the right sacrum (due to enlargement and increased activity on BS) and left posterior ilium (due to pain and local tenderness) were performed. These established the diagnoses of atypical bone island and osteoid osteoma (OO), respectively. Radiofrequency ablation of the OO resolved her symptoms. Left anterior iliac lesion was diagnosed clinically as BI. (**H**,**I**) Case 2. Hematoxylin and eosin (H&E) stain of right sacral bone biopsy, establishing a diagnosis of atypical bone island. (**H**) Magnification 10× (low power view) of the core biopsy showing cortical type bone. (**I**) Magnification 40× (high power view) of the specimen, displaying lamellar bone with small osteocytes and normal marrow components.

CT demonstrated a radiodense bone lesion with new increased uptake on BS in the left posterior ilium (Figure 4F,G), correlating with the patient’s pain, one stable centrally radiolucent lesion (atypical imaging) in the left anterior ilium (Figure 4B,E) and one enlarged faintly sclerotic lesion in the right sacrum (18 mm) when compared to five years earlier (Figure 4C,D). No BS uptake was seen in the latter two lesions. Mean CT attenuation values for these lesions were 386 HU, 1156 HU and 528 HU respectively.

The presence of left pelvic pain in association with new increased uptake on the BS in the left posterior iliac bone lesion, atypically low CT attenuation values of the left posterior iliac and right sacral lesions and enlargement of the right sacral lesion created an ambiguous clinical picture. For the bone lesion with increased uptake on BS, our differential diagnosis included osteoid osteoma, osteoblastoma, metastatic disease and atypical BI. For the enlarging bone lesion, our differential included atypical enlarging BI, low-grade central osteosarcoma, osteoblastoma and metastatic disease.

In order to clarify the situation, image guided biopsies of the left posterior iliac and right sacral lesions were performed. CT guided needle biopsy of the left posterior ilium lesion revealed woven bone formation with osteoblastic rimming, scattered osteoclasts and loose fibrovascular features consistent with osteoid osteoma (OO). Biopsy of the right sacrum showed dense laminar bone with normal marrow components, confirming a diagnosis of enostosis. The centrally radiolucent lesion in the left anterior ilium was diagnosed clinically as an atypical BI based on radiographic stability, elevated CT attenuation value consistent with BI and absence of uptake on BS. Given the patient’s history of colonic polyposis combined with osteopoikilosis, she was diagnosed with Gardner’s Syndrome. The patient’s OO was subsequently treated with microwave ablation, eliminating her symptoms.

## 4. Discussion

Since the initial description of enostosis (bone island) by Steida and Fischer in the early 20th century, there have been very few subsequent reports, likely because they are one of the most common incidentally noted bone lesions in adults [8,9]. There are rare reports of abnormal features (Table 1). However, most published studies describe only the radiologic features [2,3,4,10,11,12,13].

Enostosis, also known as bone island (BI), is a very common benign osseous lesion that consists of a focus of compact (cortical) bone within cancellous (spongy) bone [1,2]. Although the exact etiology is unknown, some authors hypothesize that BI arise due to misplaced hamartomatous cortical bone that failed to resorb during endochondral ossification [2,14]. The presence of numerous enostoses usually simply represents the condition osteopoikilosis (autosomal dominant; multiple BI; seen in two of the current cases) but rarely they may be associated with osteopathia striata (Voorhoeve’s disease), melorheostosis (unknown etiology; typified by candle wax dripping appearance of extra bone on the surface), Gardner’s syndrome (autosomal dominant; osteopoikilosis or multiple osteomas with colonic polyposis and sometimes extra-abdominal desmoid tumors and/or sebaceous cysts; the syndrome occurring in one of the current cases), Buschke-Ollendorff syndrome (autosomal dominant; osteopoikilosis and cutaneous nevi) and Gunal-Seber-Basaran syndrome (autosomal dominant; osteopoikilosis and dacrocystitis) [6,11,15,16,17,18].

Diagnosis of enostosis is based on clinical history and radiologic features. Clinically, enostoses are nearly always asymptomatic. However, there have been reports of large (>2 cm) and—rarely—small (<2 cm) painful bone islands [19,20]. Five of the patients in our cases had symptomatic enostoses and one patient had a painful osteoid osteoma simulating a BI in the setting of osteopoikilosis that mimicked an enostosis.

Enostoses are typically discovered incidentally on imaging. Plain radiographs display a homogenously dense, oval, round or oblong shaped sclerotic focus in the cancellous bone with peripheral radiating bony spicules (“rose thorn,” “thorny radiations” or “spokes”) that blend with the surrounding normal trabeculae [1,6]. On CT scan, enostoses appear as a low attenuation focus without central radiolucency and the typical peripheral radiating spicules. They must be distinguished from osteoid osteoma and osteoblastoma, which typically show a radiolucent nidus and lack the radiating spicules. On MRI enostoses display low signal intensity without surrounding edema on all signal sequences, whereas both osteoid osteoma and osteoblastoma will show extensive surrounding perilesional bone marrow edema and metastatic disease may reveal a halo of bone marrow edema surrounding the lesion [1,13]. Skeletal scintigraphy is typically “cold,” helping to distinguish it from osteoid osteoma, osteoblastoma, low-grade central osteosarcoma and most osteoblastic metastatic diseases. However, some BI have shown increased uptake on bone scan [1,12,21].

BI have a predilection for the pelvis, femur and ribs but have also been documented in the mandible, spine, carpal and tarsal bones, among other sites [6,10,14,22]. In the cases presented, sclerotic appearing enostoses were found in the pelvis, proximal and distal femur and spine in accordance with the literature.

Generally, enostoses are stable and do not typically grow in size but there have been cases of documented growth, mostly involving the mandible [4,5,14,22]. In a study of BI of the jaw, 43% detected between 9 and 19 years of age and 29% detected between 20 and 35 enlarged over time, while 5.9% diminished in size, suggesting that some BI may be labile metabolically and retain a potential for enlargement or resorption [22]. Another report describes proportional enlargement of 5 enostosis relative to bone growth in adolescents, further supporting metabolic activity in some enostoses [4].

Small, asymptomatic enostoses are managed conservatively by observation with periodic imaging. However, atypical features of enostosis such as pain, increased uptake on bone scintigraphy and enlargement mimic characteristics of active benign or malignant bone lesions, creating a differential diagnosis including osteoid osteoma, osteoblastoma, low-grade central osteosarcoma and metastatic carcinoma [6]. Atypical enostoses may pose a diagnostic challenge, potentially requiring biopsy to determine the diagnosis, as was done in seven of our cases. In one case, an osteoid osteoma in the setting of osteopoikilosis was diagnosed by biopsy.

**Figure 5:**
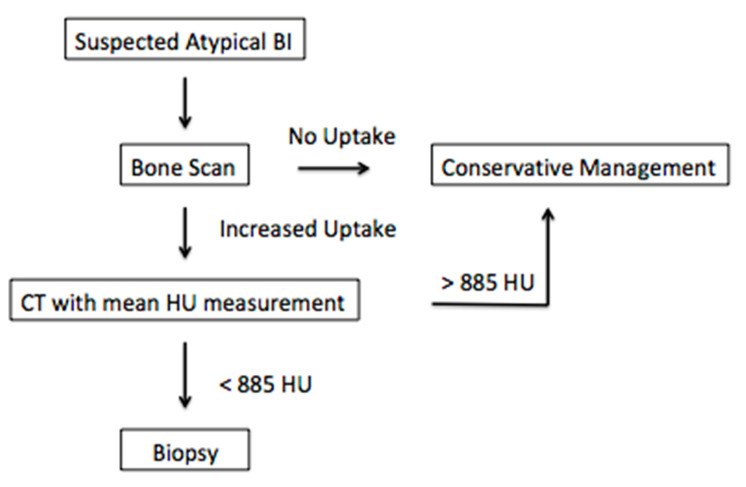
Proposed diagnostic workup of suspected atypical bone island.

Recently, specific CT attenuation thresholds have been proposed to differentiate BI from untreated osteoblastic metastasis with a mean of 885 Hounsfield Units (HU) serving as the cutoff below which a metastatic lesion is the favored diagnosis [2,3]. Nine of the ten bone islands we obtained CT attenuation values for were above this value and one was below (Table 2), providing evidence for the reliability of this threshold. Although not well established as a diagnostic tool during the evaluation of the reported series, mean HU will serve as a valuable tool in the workup of indeterminate sclerotic bony lesions, which may avoid additional invasive diagnostic measures (Figure 5).

Surgery is generally reserved for symptomatic lesions, although in cases where it is difficult to determine the source of the pain, no widely accepted guidelines exist [20]. We identified no prior publications examining HU as a means to differentiate enostoses from benign bone tumors such as osteoid osteoma, osteoblastoma or healed non-ossifying fibroma, among other possible sclerotic lesions.

## 5. Conclusions

In summary, ten patients with eleven atypical enostoses were presented, adding to the sparse literature on atypical features. Enostosis generally is considered a stable bony lesion not expected to grow in size, cause pain or show uptake on BS. However, this series, supported by review of the literature, shows that in rare cases associated pain, documented growth and even uptake on BS is seen. These findings may cause confusion with active benign or malignant bone lesions. In such instances, meticulous clinical and radiologic assessment using HU should be used with biopsy reserved for those that remain unclear. Based on our experience, reliance on mean CT attenuation values greater than 885 HU would have prevented the need for biopsy in six cases. Lastly, as illustrated by our case of osteoid osteoma simulating an enostosis in a patient with osteopoikilosis, other bone lesions may occur in the setting of osteopoikilosis, so atypical features should cause justifiable concern and appropriate action.

## Figures and Tables

**Table 1:** Clinical findings of atypical BI case reports.

Authors (Year)	# Bone Lesions	Atypical Feature
Retrospective Series		
Blank and Leiber (1965)	6	Enlargement
Onitsuka (1977)	21	Enlargement
Petrikowski and Peters (1997)	23	Enlargement
Case Reports		
Arkani et al. (1989)	1	Increased activity on nuclear medicine bone scan
Park et al. (2005)	1	Pain
Nakano et al. (2007)	1	Enlargement
Gerges et al. (2014)	1	Pain
Banzehad et al. (2020)	1	Increased activity on nuclear medicine bone scan

# = Quantity/Number of.

**Table 2:** Patient and imaging characteristics of atypical bone islands.

Case	Age (yrs)	Gender	Location	TTP	Pain	Enlargement	Typical Imaging XR/CT/MRI	CT Attenuation (HU)	Hot BS	Multiple Lesions	Rx	Final Dx
1	46	F	Posterior ilium (L)	N	N	Y	Y	998	N	Y	Obs	OP; AEBI
2	43	F	Sacrum (R)	N *	N ^^^	Y	N	528	N	Y	Bx: BI	OP; GS; AEBI
			Anterior ilium (L)	N ^#^	Y	N	N (central lucency)	1156	N	Y	Obs	OP; GS; ABI
			Posterior ilium (L)	Y	Y	N	Y	386	Y	Y	Bx: OO (RFA)	OP; GS; OO
3	80	M	Posterior ilium (R)	N	N	Y	Y	1082	Y	N	Obs	AEBI
4	59	F	Pubic body (R)	Y	Y	Y	N (periph. Cortical erosion)	1196	N	N	Bx: BI	AEBI
5	60	F	Femoral neck (L)	N	N	Y	Y	1002	N	N	Bx: BI	AEBI
6	64	F	Acetabulum (R)	N	N	Y	Y	1001	N	N	Obs	AEBI
7	57	F	L3 vertebrae (L)	N	N	N	Y	1375	Y	N	Bx: BI	ABI
8	47	F	Anterior ilium (L)	N	Y	N	Y	1015	N	N	Bx: BI	ABI
9	46	F	Femoral head/neck (R)	N	Y	Y	Y	1229	Y	N	Bx: BI	AEBI
10	87	M	Femoral distal medial condyle (R)	N	Y	N	Y	Not available ^+^	Y	N	Bx: BI	ABI

Yrs = years; F = female; M = male; TTP = tenderness to palpation; Y = yes; N = no; Rx = management; XR = X-ray; CT = computerized tomography; MRI = magnetic resonance imaging; HU = hounsfield units; BS = nuclear medicine bone scintigraphy; Hot BS = increased uptake at site of lesion in question; R = right; L = left; Obs = observation; Bx = biopsy; OP = osteopoikilosis; GS = Gardner’s syndrome; AEBI = atypical enlarging bone island; ABI = atypical bone island; RFA = radiofrequency ablation; * NTTP over site of atypical lesion but TTP on contralateral side; ^^^ Pain contralateral pelvis only; ^#^ TTP posterior but not anterior; ^+^ No pre-operative CT performed.
